# Synergistic Effects of Autologous Plasma and Agarose Fillers in Treating Acne, Atrophic, and Hypertrophic Scars: A Case Series

**DOI:** 10.1093/asjof/ojaf130

**Published:** 2025-10-17

**Authors:** Marwa Zein AlAbidden, Joan Vandeputte

## Abstract

**Background:**

Scars are a common outcome of wound healing, affecting millions worldwide. These marks can result from various causes. However, current treatments, such as dermal fillers and laser therapy, often have limitations, including transient effects that do not stimulate the body's natural regenerative processes, high costs, and potential adverse reactions.

**Objectives:**

The objective of this study is to explore the potential therapeutic synergistic effect of a novel combination therapy using agarose and autologous platelet-rich plasma (PRP) to improve the appearance and characteristics of acne, atrophic, and hypertrophic scars.

**Methods:**

This retrospective case series included 8 patients with acne, atrophic, and hypertrophic scars. All participants received a 50/50% mixture of agarose 1% and autologous PRP injected into the affected areas. Acne scars were evaluated using the Goodman and Baron Qualitative Acne Scar Grading System and the Investigator Global Acne Scarring (IGAS) scale. Atrophic and hypertrophic scars were assessed using the Modified Scar Assessment Scale (MSAS) and Investigator Global Aesthetic Improvement Scale (IGAIS). Patient satisfaction for all scar types was measured using the Patient Satisfaction Scale (PSS) at the final follow-up.

**Results:**

Combined agarose–PRP therapy demonstrated clinical improvement in all treated scars. Acne scar patients (*n* = 2) showed a mean improvement of 2 points on the Goodman and Baron scale and the IGAS. For atrophic scar patients (*n* = 6), the MSAS scores improved from Grade 3-4 to Grade 0-1. IGAIS scores ranged from +2 to +3. PSS was high, with most patients reporting being “very satisfied” or “moderately satisfied.” Representative images show visible improvements in scar depth, texture, and overall appearance.

**Conclusions:**

Agarose–PRP filler shows promise for scar treatment. Physicians should be aware that these findings may represent an effective treatment option for patients. These results may be helpful in guiding treatment decisions for scar management.

**Level of Evidence: 4 (Therapeutic):**

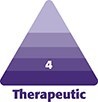

Atrophic scars, whether caused by traumatic injuries, chickenpox, improperly healed surgical wounds, or acne, can significantly impact an individual's quality of life, affecting not just appearance but also function and self-perception.^1^ Insufficient collagen production during healing results in atrophic scars.^2^ These sunken or indented marks are prevalent among acne patients, with over 87% of individuals with mild-to-moderate acne experiencing some degree of scarring.^3^ Atrophic scars are classified into 3 main types based on their shape and depth: ice-pick scars, which are narrow and deep, boxcar scars, which are broader with sharply defined edges and rolling scars, which have a wave-like appearance because of tethering of the skin to underlying structures.^4^ The appearance of atrophic scars can vary depending on factors such as scar location and age.^5^ These scars are particularly challenging to treat because of the loss of dermal tissue, which makes restoring volume and texture difficult.^4^

Current therapeutic modalities for scar management, while providing some degree of improvement, are often limited by challenges such as inconsistent results or safety concerns.^6,7^ In contrast, agarose, an agarose-based injectable filler used in this study, offers innovative and distinctive characteristics that address these limitations, making it a valuable advancement in aesthetic medicine. The polysaccharide in agarose forms a particle gel with a microporous structure, effectively restoring volume lost because of aging, enhancing facial contours, and smoothing wrinkles and folds.^8-10^ Unlike hyaluronic acid (HA) fillers that rely on cross-linking agents like BDDE or PEG, agarose integrates well into tissues, demonstrating excellent biodegradability and a reduced risk of adverse reactions.^11^ Autologous plasma, specifically platelet-rich plasma (PRP), is becoming widely used at the forefront of regenerative technologies. The primary advantage of using autologous plasma is its biocompatibility and safety.^12^ Consequently, this makes PRP highly attractive for a range of medical applications, including orthopedics, dermatology, and wound healing.^13^ The composition of plasma plays a critical role in numerous physiological functions, particularly in tissue regeneration.^14^

A new therapy that combines agarose fillers with autologous plasma stands out because of the unique properties of the components. Agarose fillers provide a biocompatible scaffold that resembles the extracellular matrix, supporting tissue regeneration, maintaining structural integrity, and preventing irregular scar formation.^15^ Additionally, autologous plasma enriched with growth factors and cytokines increases collagen production and accelerates healing.^16^ In the current study, we aim to employ this innovative combination therapy, harnessing its synergistic effects to enhance these mechanisms for long-term scar management.

## METHODS

### Patient Selection and Study Design

This retrospective case series aimed to evaluate the efficacy of agarose-based injectable filler combined with autologous PRP for treating atrophic scars. The study included patients with atrophic scars, including both posttraumatic and acne scars. A total of 8 patients were enrolled, categorized into 2 groups: acne-related atrophic scars (2 patients) and posttraumatic or surgical atrophic scars (6 patients). Distinct treatment protocols were applied based on the type of scar. A summary of the patient demographics, including age, sex, scar type, cause, location, and specific scar features, is provided in Table 1.

**Table 1.** Patient Demographics and Scar Characteristics

Patient no.	Age (years)	Sex	Scar type	Cause	Location	Scar characteristics
1	26	F	Acne scars (atrophic)	Postinflammatory acne	Cheeks	Enlarged pores, no active inflammation
2	59	M	Acne scars (ice-pick, atrophic, fibrotic)	Chronic acne	Face, submandibular neck	Multiple small atrophic scars, retracted areas, large pores
3	26	F	Linear atrophic post 50/50 triamcinolone–5-FU	Glass laceration	Décolletage	Stretched, raised centrally, pink-red, some atrophic areas
4	36	F	Depressed linear atrophic scar	Achilles tendon surgery	Right Achilles tendon	Longstanding atrophic scar
5	59	M	Linear atrophic scar	Crush injury (marble plinth)	Left pretibial area	Thin dermis, dry, scaly, light pink, hyperpigmentation
6	34	F	Linear atrophic scar	Beirut port explosion trauma	Left temporal region	Posttraumatic linear atrophic scar with fibrotic remodeling, pigmentary alterations, deep atrophy, contour depression, and asymmetry
7	45	F	Mixed atrophic-hypertrophic scar	Beirut port explosion trauma	Décolleté area	Mixed posttraumatic scar with fibrotic remodeling, deep atrophy, hypertrophic thickening, textural irregularities, hypopigmentation, and redness
8	37	F	Linear atrophic scar	Beirut port explosion trauma	Mandibular area	Mild atrophy, subtle contour irregularity, textural differences

“F” refers to female and “M” refers to male. Scars are categorized into acne-related atrophic and posttraumatic or surgical atrophic scars.

### Inclusion and Exclusion Criteria

Inclusion criteria required the presence of atrophic scars (linear, rolling, boxcar, or ice-pick), or mixed scars with atrophic and fibrotic components. Scars had to be ≥6 months old, stable, and not in an active inflammatory or growth phase. Only patients aged 18 to 65 years in good general health were included. The scars should have caused aesthetic or psychosocial distress, with no active acne or dermatoses at the treatment site. Patients were required to undergo up to 3 sessions of PRP–agarose injections, spaced 1 month apart, and commit to a 6-month follow-up period. Signed informed consent was obtained from all participants.

Exclusion criteria included active keloids or unstable hypertrophic scars, scars younger than 6 months, ulcerated, infected, or ischemic scars, a history of platelet dysfunction syndromes or coagulopathies, autoimmune dermatologic conditions, or current use of immunosuppressive medications. Patients with known hypersensitivity to agarose or PRP components, pregnancy or breastfeeding, or unrealistic expectations regarding the outcome of treatment were excluded. Recent aesthetic procedures within the treatment area (in the past 3 months) were also grounds for exclusion. Patients were explicitly instructed not to use any other topical creams, lotions, chemical peels, lasers, or additional scar-targeted treatments during the entire study duration and follow-up period. Compliance with these instructions was confirmed clearly at each follow-up visit.

### Treatment Protocol

#### Platelet-Rich Plasma Preparation

After thorough skin disinfection with chlorhexidine 0.5% in alcohol, 18 mL of venous blood was drawn into a 20 mL syringe containing 2 mL of citrate buffer (trisodium citrate 3.3 mg/mL, citric acid 1.2 mg/mL, dextrose 3.68 mg/mL). The blood was transferred into a DrPRP Platelet-Rich Plasma Separator and centrifuged at 3200 RPM for 3 to 4 min to isolate the buffy coat. PRP was aspirated into sterile syringes, with the volume prepared varying according to the treatment requirements.

#### Agarose Gel Preparation

Agarose 1% (Algeness LD%, Advanced Aesthetic Technologies, One Brookline Place, Suite 427, Brookline, MA) was provided in prefilled 1.4 mL syringes. Equal volumes of PRP and agarose gel were mixed by repeatedly flushing the solution between 2 sterile syringes until a homogeneous consistency was achieved (Video 1). The final mixture was prepared in multiple syringes for injection.

#### Injection Protocol

For atrophic scars, the PRP–agarose combination was injected subdermally using a 25G 40 mm cannula in linear retrograde passes (Videos 2, 3 show the injection methods for Patients 3 and 4). Manual pressure was applied beside the scar to minimize filler spread into surrounding tissue. Local anesthesia was administered as needed, and the procedure was repeated in 3 sessions, spaced 1 month apart.

For acne scars, a similar PRP–agarose combination was used but with modifications tailored to acne-related atrophy. The injections were more localized to address deeper scars and improve volume. Intradermal micro-droplet injections using a 30G needle were used for ice-pick scars and enlarged pores, while subdermal injections with a 25G 40 mm cannula and a 27G needle focused on lifting retracted and atrophic regions. Linear retrograde threading was used to detach fibrotic scar tissue and improve overall skin texture (Videos 4 show the injection methods for Patient 2, including subdermal and intradermal injections, as well as local anesthesia administration). Treatment was adjusted based on patient response and follow-up assessments to optimize scar remodeling. Of the 2 cases, Patient 2 (a 59-year-old male with ice-pick, atrophic, and fibrotic acne scars because of chronic acne) underwent a single session of submandibular neck superficial and subcutaneous injections for the face. This treatment was deemed sufficient for this particular patient. Following injections, manual pressure and massaging techniques were applied to ensure even distribution of the filler and enhance integration into the scarred tissue.

Injection volumes were individually determined based explicitly on scar size, depth, severity, and anatomical location, aiming to adequately fill scars without causing excessive filler spread or palpable irregularities. The goal was to achieve optimal scar leveling and volume restoration, with careful manual pressure and massaging techniques to ensure uniform distribution and integration.

#### Outcome Measures

Linear atrophic scar outcomes were evaluated using 3 scales, including the Modified Scar Assessment Scale (MSAS) for objective assessment of pigmentation, vascularity, pliability, texture, and overall appearance; the Investigator Global Aesthetic Improvement Scale (IGAIS) for clinician-reported scar improvement; and the Patient Satisfaction Scale (PSS) for patient-reported satisfaction. For acne scars, severity was evaluated with the Goodman and Baron Qualitative Acne Scar Grading System, which categorizes scars into macular, mild, moderate, and severe grades based on color changes, texture, and resistance to stretching, while the Investigator Global Acne Scarring (IGAS) evaluates acne scar severity on a 4-point scale, ranging from clear skin (Grade 1) to severe scarring (Grade 4), based on the overall appearance and depth of scars. Patient satisfaction was measured using the PSS at the final follow-up. Results were assessed at baseline and at the 6-month follow-up to evaluate the effectiveness of the treatment.

## RESULTS

### Acne Scar Treatment Results (*n* = 2)

**Figure 1.**
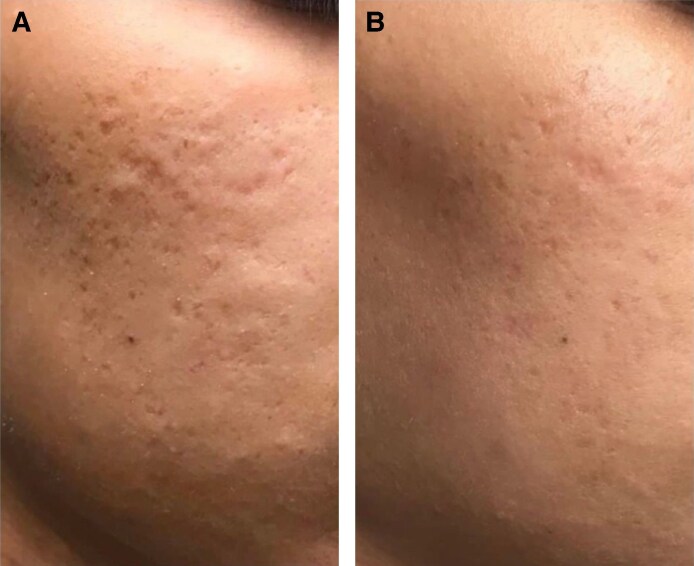
(A) Patient 1: a 26-year-old female with Grade 3 acne scars before treatment. Pretreatment photograph shows visible indentations, hyperpigmentation, and enlarged pores (Goodman and Baron Scale: Grade 3). (B) Posttreatment photograph of Patient 1, taken 6 months after Algeness platelet-rich plasma therapy. Demonstrates marked improvement (Investigator Global Aesthetic Improvement Scale +2, Goodman and Baron Scale improved to Grade 1) with reduced scar depth, improved skin texture, and decreased pore size. The patient reported being very satisfied (Patient Satisfaction Scale: 4/5).

**Figure 2.**
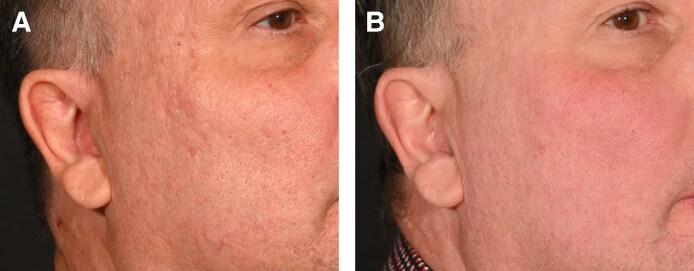
(A) Patient 2: a 59-year-old male with Grade 3 acne scars before treatment. Pretreatment photograph shows visible atrophic scars, enlarged pores, and irregular skin texture (Goodman and Baron Scale: Grade 3). (B) Posttreatment photograph of Patient 2, taken 6 months after Algeness platelet-rich plasma therapy. Demonstrates marked improvement (Investigator Global Aesthetic Improvement Scale +2, Goodman and Baron Scale improved to Grade 1) with reduced scar depth, smoother skin texture, and smaller pore size. The patient reported being very satisfied (Patient Satisfaction Scale: 4/5).

Two patients with moderate (Grade 3) acne scarring were treated with combined agarose–PRP therapy. Patient 1, a 26-year-old female, presented with ice-pick and rolling scars (Figure 1A), while Patient 2, a 59-year-old male, exhibited predominantly rolling scars with mild boxcar components (Figure 2A). Both patients showed marked improvement (IGAS +2) at follow-up (6 months posttreatment). The Goodman and Baron Qualitative Scale improved from Grade 3 (moderate) to Grade 1 (mild) in both patients. Patient 1 demonstrated significant reduction in ice-pick scar depth and rolling scar visibility, with improved skin texture, diminished hyperpigmentation, and decreased pore size. Clinical examination revealed improved dermal thickness and hydration, with less tethering to underlying structures (Figure 1B). Similarly, Patient 2 showed noticeable reduction in rolling scar depth, moderate improvement in boxcar scars, decreased pore size, and reduced postinflammatory erythema, resulting in a more uniform skin tone (Figure 2B). Both patients reported high satisfaction (PSS: 4/5), indicating major visible improvement with only minor residual textural irregularities (Table 2).

**Table 2.** Summary of Clinical Outcomes Following Treatment

Patient	Scar type(s)	Pretreatment score	Posttreatment score	IGAS	PSS
1	Ice-pick and rolling scars	Goodman and Baron Grade 3 (moderate)	Goodman and Baron Grade 1 (mild)	+2 (marked improvement)	4/5 (very satisfied)
2	Predominantly rolling scars with Mild boxcar	Goodman and Baron Grade 3 (moderate)	Goodman and Baron Grade 1 (mild)	+2 (marked improvement)	4/5 (very satisfied)

Scar types, pre- and posttreatment scores of acne scars, based on the Goodman and Baron Qualitative Scale, IGAS, and PSS, are shown. IGAS, Investigator Global Acne Scarring; PSS, Patient Satisfaction Scale.

### Atrophic and Hypertrophic Scar Treatment Results (*n* = 6)

**Figure 3.**
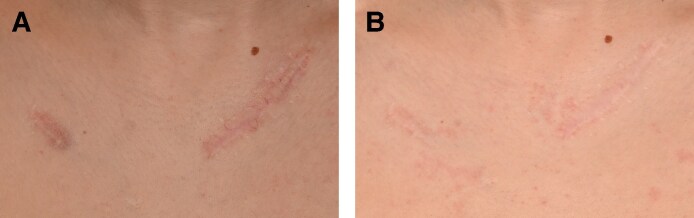
(A) Patient 3: a 26-year-old female with linear atrophic and hypertrophic scars on the décolletage and right breast following a glass laceration. Pretreatment photograph shows stretched, raised scars centrally, pink-red discoloration, with some atrophic areas (Modified Scar Assessment Scale [MSAS] Grade 4: severe). (B) Posttreatment photograph of Patient 3, taken 6 months after Algeness platelet-rich plasma therapy. It demonstrates marked improvement (Investigator Global Aesthetic Improvement Scale +2, MSAS Grade improved to Grade 1), including reduced scar elevation, smoother texture, and decreased redness. The patient reported being very satisfied (Patient Satisfaction Scale: 4/5).

**Figure 4.**
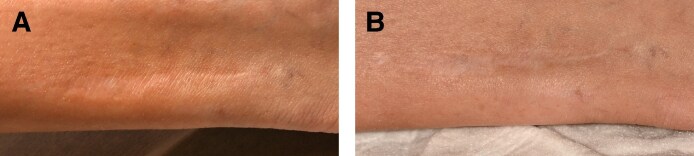
(A) Patient 4: a 36-year-old female with a longstanding depressed linear atrophic scar following Achilles tendon surgery. Pretreatment photograph shows a visible scar with significant depth and texture irregularities (Modified Scar Assessment Scale [MSAS] Grade 4: Severe). (B) Posttreatment photograph of patient No. 4, taken 6 months after Algeness platelet-rich plasma therapy. It demonstrates marked improvement (Investigator Global Aesthetic Improvement Scale +2, MSAS Grade improved to Grade 1), including reduced scar depth and smoother texture. The patient reported being very satisfied (Patient Satisfaction Scale: 4/5).

**Figure 5.**
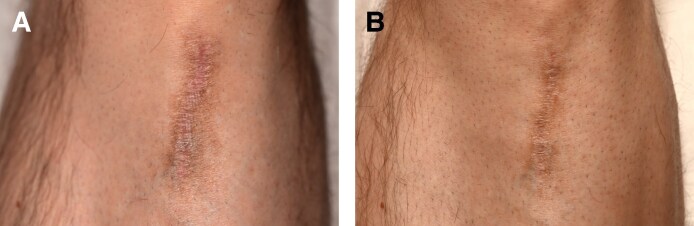
(A) Patient 5: a 59-year-old male with a linear atrophic scar on the left pretibial area resulting from a crush injury. Pretreatment photograph shows a severe scar (Modified Scar Assessment Scale [MSAS] Grade 4) with thin dermis, dry and scaly texture, light pink discoloration, and hyperpigmentation. (B) Posttreatment photograph of Patient 5, taken 6 months after Algeness platelet-rich plasma therapy. It demonstrates marked improvement (Investigator Global Aesthetic Improvement Scale +2, MSAS improved to Grade 1), including reduced atrophy, improved skin texture, and overall enhanced appearance. The patient reported being very satisfied (Patient Satisfaction Scale: 4/5).

Posttraumatic and surgical atrophic scars showed significant improvement with the treatment. Six patients (4 females, 2 males; age range, 26-59 years) with various atrophic scars were treated, including patients resulting from glass laceration (Patient 3, Figure 3A), Achilles tendon surgery (Patient 4, Figure 4A), crush injury (Patient 5, Figure 5A), and Beirut port explosion trauma (Patients 6-8). Before the treatment, the scars displayed characteristics such as moderate-to-severe atrophy, fibrotic remodeling, pigmentary changes, and uneven textures. Following 3 treatment sessions, 6-month follow-up assessments revealed marked improvements across all patients.

**Figure 6.**
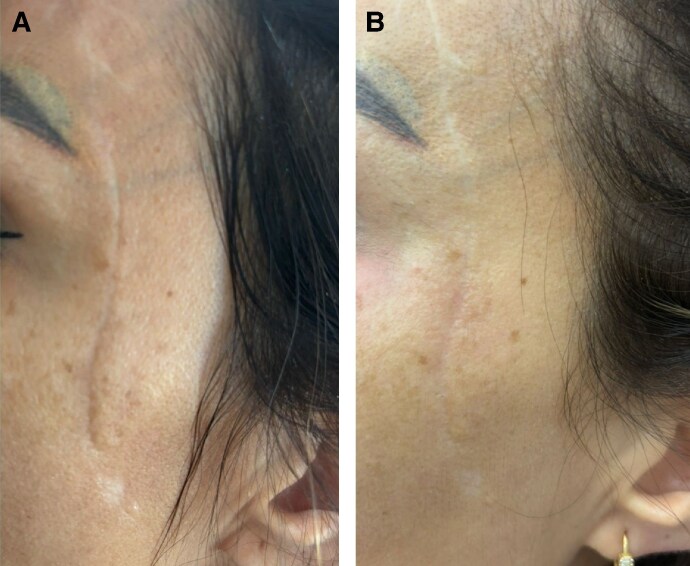
(A) Patient 6: a 34-year-old female with a posttraumatic linear atrophic scar on the left temporal region resulting from the Beirut port explosion. The pretreatment photograph shows a severe scar (Modified Scar Assessment Scale [MSAS] Grade 4) characterized by fibrotic remodeling, pigmentary alterations, deep atrophy, contour depression, and asymmetry. (B) Posttreatment photograph of Patient 6, taken 6 months after Algeness platelet-rich plasma therapy. It demonstrates marked improvement (Investigator Global Aesthetic Improvement Scale +2, MSAS improved to Grade 1) including significant reduction in atrophy and enhanced skin texture. The patient reported being very satisfied (Patient Satisfaction Scale: 4/5).

**Figure 7.**
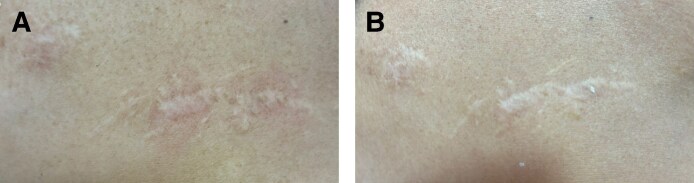
(A) Patient 7: a 45-year-old female with a mixed atrophic-hypertrophic scar on the décolleté area resulting from the Beirut port explosion trauma. The pretreatment photograph reveals a complex posttraumatic scar (Modified Scar Assessment Scale [MSAS] Grade 3) characterized by fibrotic remodeling, deep atrophy, hypertrophic thickening, textural irregularities, hypopigmentation, and redness. (B) Posttreatment photograph of Patient 7, taken 6 months after Algeness platelet-rich plasma therapy. It demonstrates significant improvement (Investigator Global Aesthetic Improvement Scale +2, MSAS improved to Grade 1) including marked reduction in atrophy and fibrosis, improved skin contour, and near-resolution of erythema. The patient reported being very satisfied (Patient Satisfaction Scale: 4/5).

**Figure 8.**
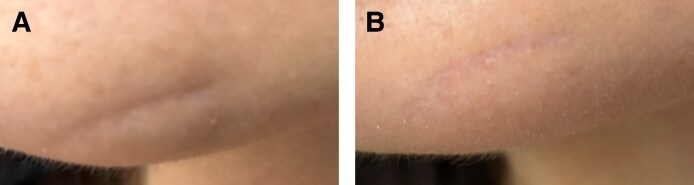
(A) Patient 8: a 37-year-old female with a linear atrophic scar on the mandibular area resulting from the Beirut port explosion trauma. The pretreatment photograph shows a mild atrophic scar with subtle contour irregularity and textural differences. (B) Posttreatment photograph of Patient 8, taken 6 months after Algeness platelet-rich plasma therapy. It demonstrates near-complete resolution (Investigator Global Aesthetic Improvement Scale +3, Modified Scar Assessment Scale improved to Grade 0). The image reveals exceptional improvement, indicating no visible scar. The patient reported being very satisfied (Patient Satisfaction Scale: 4/5).

Patient 3 presented with a combination of hypertrophic and atrophic linear scars, characterized by raised central areas and peripheral thinning, particularly on the décolletage and right breast (Figure 3A). Following treatment, there was a notable improvement in both scar elevation and contour depression, reflecting a reduction in the hypertrophic and atrophic components, respectively (Figure 3B). Patient 4 showed improved surface regularity and minimal residual depression (Figure 4B). Patient 5 exhibited restored contour symmetry and more uniform pigmentation (Figure 5B). Across all 3 patients, MSAS scores improved from Grade 4 (severe) to Grade 1 (mild), with IGAIS scores of +2 (marked improvement), and Patient Satisfaction Scores were consistently rated 4/5 (Table 3). Patients 6 to 8, all resulting from the Beirut port explosion, showed a spectrum of improvements. Patient 6 (Figure 6A) exhibited improved dermal thickness and reduced hyperpigmentation, although some pigmentary alterations remained (MSAS improved from Grade 4 to Grade 1, IGAIS +2; Figure 6B). Patient 7 (Figure 7A) showed a significant reduction in atrophy and fibrosis and improvement in skin contour, with erythema nearly resolved (MSAS improved from Grade 3 to Grade 1, IGAIS +2; Figure 7B). Patient 8 (Figure 8A) achieved near-complete resolution with exceptional improvement (IGAIS +3) and an MSAS score of 0, indicating no visible scar (Figure 8B). PSS ranged from moderately to very satisfied (3-4/5) across these patients (Table 3).

**Table 3.** Summary of Clinical Outcomes Following Treatment

Patient	Scar type	Pretreatment score	Posttreatment score	IGAIS	PSS
3	Linear atrophic	MSAS Grade 4 (severe)	MSAS Grade 1 (mild)	+2 (marked improvement)	4/5 (very satisfied)
4	Depressed linear atrophic	MSAS Grade 4 (severe)	MSAS Grade 1 (mild)	+2 (marked improvement)	4/5 (very satisfied)
5	Linear atrophic	MSAS Grade 4 (severe)	MSAS Grade 1 (mild)	+2 (marked improvement)	4/5 (very satisfied)
6	Linear atrophic	MSAS Grade 4 (severe)	MSAS Grade 1 (mild)	+2 (marked improvement)	4/5 (very satisfied)
7	Mixed atrophic-hypertrophic	MSAS Grade 3 (severe)	MSAS Grade 1 (mild)	+2 (marked improvement)	3/5 (moderately satisfied)
8	Linear atrophic	MSAS Grade 2 (moderate)	MSAS Grade 0 (none)	+3 (exceptional improvement)	4/5 (very satisfied)

Scar type, pre- and posttreatment scores of atrophic and hypertrophic scars based on the MSAS, IGAIS, and PSS are shown. IGAIS, Investigator Global Aesthetic Improvement Scale; MSAS, Modified Scar Assessment Scale; PSS, Patient Satisfaction Scale.

In summary, combined agarose–PRP therapy resulted in significant improvements in posttraumatic and surgical atrophic scars. Across all 6 patients, MSAS scores improved from Grade 2-4 (moderate-severe) to Grade 0-1 (none-mild), IGAIS scores ranged from +2 (marked improvement) to +3 (exceptional improvement), and PSS scores were consistently high (3-4/5), indicating a high degree of patient satisfaction (Table 3).

## DISCUSSION

Agarose, an organic filler derived from purified red algae, is sterilized for human injection without undergoing chemical modification.^17^ It is completely resorbable in situ by a histiocytary infiltrate without capsule formation.^8^ It is a particle gel, commercially available in 4 concentrations (1%, 1.5%, 2.5%, and 3.5%). The 2.5% and 3.5% concentrations are formulated with 0.5% and 0.4% noncross-linked HA, respectively, to enhance injection smoothness. It is principally to be injected subcutaneously or deep, because the histiocytary infiltrate is sometimes palpable, especially for the highest of the 4 commercially available concentrations. Agarose particles are porous, which can be visualized by electron microscopy.^18^ They are saturated with water and do not take up water after injection nor induce water retention in the surrounding tissues in the mid or long term. The injection of PRP has been shown to accelerate healing, improve delayed healing, enhance scar maturation, improve hair growth, and improve the quality of injected skin and mucous membranes.^19^

The results of this study demonstrate that combined agarose–PRP therapy is a promising treatment modality for both acne and atrophic scars. In the treatment of acne scars, the significant improvements observed in both patients, as measured by the Goodman and Baron and IGAS scales, suggest that agarose–PRP effectively addresses multiple aspects of acne scarring. The reduction in ice-pick and rolling scar depth, improved skin texture, diminished hyperpigmentation, and decreased pore size highlight the potential of this combined approach to stimulate collagen synthesis, promote dermal remodeling, and improve overall skin quality. The increased dermal thickness and hydration noted in Patient 1, coupled with the reduced tethering to underlying structures, suggest that agarose–PRP may help to restore the structural integrity of the skin and release fibrotic adhesions that contribute to scar formation.

The reduction in rolling scar depth, improvement in boxcar scars, decreased pore size, and reduced postinflammatory erythema observed in Patient 2 further support the versatility of this treatment for addressing various types of acne scars. These results align with several studies that have shown the effectiveness of both plasma therapy and afillers in treating scars and stimulating collagen production during skin regeneration. A comprehensive review discussing the uses of PRP emphasizes its ability to boost collagen synthesis and accelerate wound healing by releasing important growth factors, such as platelet-derived growth factor, insulin-like growth factor, and transforming growth factor beta. These growth factors play a crucial role in promoting cell growth and differentiation, essential for tissue recovery, ultimately minimizing scar formation.^12^ Furthermore, it has been established in clinical studies that agarose gives an immediate volumetric effect that is safe, predictable, and long-lasting results. Also, it was reported that the use of agarose is very useful in cosmetic and reconstructive procedures on the face and neck.^8^

The results of combined agarose–PRP therapy in treating both atrophic and hypertrophic scars were very promising. Notably, the 50/50% agarose 1%/PRP mixture yielded remarkable results in hypertrophic scars that transitioned to an atrophic state after initial treatments, as highlighted in Patient 3. Subsequent injections reduced the hardness and redness of the scars, ultimately leveling them with the surrounding skin and achieving minimal pigmentation and consistent firmness with healthy tissue or mature atrophic scars. In Patients 4 and 5, the therapy improved the texture and consistency of the scars, rendering them soft, pale, and level with the surrounding skin. These treated scars showed no further depression or hardness, blending seamlessly with the surrounding tissue.

In patients involving atrophic scars in the maturation phase, particularly those resulting from blast trauma during the Beirut port explosion (Patients 6-8), varying levels of improvement were observed. The therapy notably addressed dryness, atrophy, and pink coloration, leading to less depressed scars, reduced hyperpigmentation, and increased dermal thickness over repeated treatments. The absence of nodules or firmness with the 1% PRP–agarose (50/50) mix highlights its safety profile—particularly because higher concentrations of agarose can result in palpable subdermal infiltrates or nodularity. Exceptional improvement was achieved in Patient 8, with near-complete resolution of the scars. These findings highlight the versatility and effectiveness of the PRP–agarose combination in treating diverse types of scarring, likely through complementary mechanisms, such as collagen synthesis, dermal remodeling, and hydration enhancement. These mechanisms are supported by several studies, including a research study comparing agarose gel and collagen in a rat model found that agarose gel showed significant biological activity and promoted new collagen formation without negative reactions, highlighting the potential of agarose in tissue repair.^20^

Moreover, clinical trials have proven that using agarose fillers leads to immediate and long-lasting effects for both cosmetic and reconstructive procedures. These fillers compete well with HA-based materials and deliver natural-looking results. They integrate smoothly with tissues, reducing scarring and promoting wound healing and collagen production by maintaining a moist environment and aiding in cell movement.^7,8,11,21,22^ Beyond that, the effectiveness of 2.5% agarose gel was compared with HA fillers for treating moderate-to-severe nasolabial folds. Results demonstrated that agarose provides comparable or even superior outcomes in volume restoration, attributed to its high elasticity and viscosity.^11^ Patients treated with agarose reported high levels of satisfaction and a natural appearance posttreatment.^11^ Agarose poses a lower risk of adverse reactions, as agarose does not induce mid- or long-term edema, and maintains an improved shape over time. Unlike HA fillers, agarose cannot be enzymatically dissolved; however, it undergoes full resorption through a histiocytic infiltrate, as demonstrated in animal studies. This clearance mechanism offers a predictable degradation profile, although the absence of a dissolving agent may limit immediate reversibility.^9^

In addition to the benefits of agarose fillers, PRP therapy further supports regenerative processes. PRP therapy sessions, which utilize autologous plasma, are supported by research into megakaryocytes and platelets derived from iPSCs, demonstrating their effectiveness in accelerating wound healing and promoting collagen production.^23^ These cells release growth factors that significantly boost wound healing and collagen synthesis, highlighting their potential in regenerative medicine. Together with cytokines and adhesion molecules like fibrinogen and fibronectin, these components accelerate tissue repair processes by supporting cell migration and extracellular matrix synthesis. This composition makes PRP an effective tool in regenerative medicine for promoting tissue repair and functional recovery across various medical fields.^12^

Upon detailed evaluation of scar response across cases, we noted particularly noticeable improvement in acne-related atrophic scars (rolling and ice-pick types), likely because of the combined volume restoration and regenerative stimulation by PRP–agarose injections. Linear posttraumatic atrophic scars also responded very consistently, demonstrating substantial improvement in skin texture and contour. These differences could relate to scar depth, underlying fibrosis, scar maturity, and biological responses to agarose and growth factors present in PRP. Future larger comparative studies specifically designed to analyze the differential response among distinct scar types would clarify these preliminary observations and guide targeted clinical protocols.

On the other hand, scar location and injection volume are critical technical factors influencing clinical outcomes. Larger scars, particularly those spanning broad surface areas or located in areas of high skin tension, could potentially benefit from higher PRP–agarose injection volumes. This increased volume might provide enhanced structural support, help mitigate skin tension forces more effectively, and promote more uniform dermal remodeling and scar leveling. However, caution is required to avoid excessive filler spread, uneven filler distribution, or palpable irregularities. Future controlled trials are warranted to explicitly investigate and optimize the relationship between injection volumes, scar sizes, anatomical locations, and tension forces to improve scar treatment protocols further.

Although the improvements we observed were attributed to the combined effect of PRP and agarose, we acknowledge that part of the clinical outcome may be related to the mechanical action of the needle itself. The act of subcision, used in some of the cases, might have contributed to collagen stimulation and improved scar mobility. This raises an important question: how much of it comes from the injectable, and how much from the technique? A comparative study between simple subcision vs PRP–agarose injection would help clarify this. It is something we plan to explore in future controlled trials, ideally with a larger number of patients and longer-term follow-up.

A limitation of our study is the lack of long-term follow-up, which prevents definitive conclusions about the durability of treatment outcomes. Additionally, these are noncomparative case reports spanning diverse scar types and anatomical sites, limiting generalizability and precluding direct comparisons. Nonetheless, the consistent clinical improvements observed suggest a potential role for this approach within regenerative medicine.

## CONCLUSIONS

In conclusion, the use of a 50/50% mixture of agarose 1% and PRP has proven to be an effective and synergistic treatment for various types of atrophic scars. It improves scar texture, consistency, and appearance, demonstrating significant potential as a safe and reliable therapeutic approach with no notable adverse effects. Further research and comparative studies with other treatment modalities are essential to fully establish its efficacy and optimize its application in clinical practice.
